# *CNTN-1* Upregulation Induced by Low-Dose Cisplatin Promotes Malignant Progression of Lung Adenocarcinoma Cells *via* Activation of Epithelial-Mesenchymal Transition

**DOI:** 10.3389/fgene.2022.891665

**Published:** 2022-05-27

**Authors:** Ruijie Zhang, Shengjin Li, Jian Lan, Changyi Li, Xianzhi Du, Weijie Dong, Qian Yu, Daoxin Wang

**Affiliations:** Department of Respiratory and Critical Care Medicine, The Second Affiliated Hospital of Chongqing Medical University, Chongqing, China

**Keywords:** CNTN-1, EMT, cisplatin, A549 cell, metastasis, invasion

## Abstract

Tumor metastasis and invasion are the main impediments to lung adenocarcinoma successful treatment. Previous studies demonstrate that chemotherapeutic agents can elevate the malignancy of cancer cells other than their therapeutic effects. In this study, the effects of transient low-dose cisplatin treatment on the malignant development of lung adenocarcinoma cells (A549) were detected, and the underlying epigenetic mechanisms were investigated. The findings showed that A549 cells exhibited epithelial-mesenchymal transition (EMT)-like phenotype along with malignant progression under the transient low-dose cisplatin treatment. Meanwhile, low-dose cisplatin was found to induce contactin-1 (*CNTN-1*) upregulation in A549 cells. Subsequently, we found that further overexpressing *CNTN*-1 in A549 cells obviously activated the EMT process *in vitro* and *in vivo*, and caused malignant development of A549 cells *in vitro*. Taken together, we conclude that low-dose cisplatin can activate the EMT process and resulting malignant progression through upregulating *CNTN-1* in A549 cells. The findings provided new evidence that a low concentration of chemotherapeutic agents could facilitate the malignancy of carcinoma cells *via* activating the EMT process other than their therapeutic effects.

## Introduction

Non–small cell lung cancer (NSCLC) is one of the leading causes of mortality in the world, and lung adenocarcinoma is identified as the most common pathological type of NSCLC (Corral de la Fuente et al., 2021; Coleman et al., 2022). Though considerable manpower and research funds are applied to explore the mechanism of tumor formation and progression, the treatment efficiency for advanced lung adenocarcinoma is still unsatisfying. It is well established that tumor metastasis and invasion are the main impediments to tumor successful treatment, which finally lead to a dismal prognosis of lung adenocarcinoma (Gong et al., 2021). A comprehensive understanding of the underlying molecular mechanisms of lung adenocarcinoma progression will help to establish effective therapies.

Epithelial-mesenchymal transition (EMT) is a dynamic cellular reprogramming process through which epithelial cells undergo morphological changes characterized by loss of cell-cell adhesion and acquisition of mobile fibroblast-like phenotype. A previous study demonstrated that EMT progression participated in embryonic development and tissue remolding (Greaves and Calle, 2022). Subsequent evidence suggested that the aberrant activation of the EMT process promoted metastasis, invasion, and drug resistance of multiple epithelial carcinomas, such as gastric carcinoma, breast cancer, and lung cancer (Menju and Date, 2021; Azimi et al., 2022; Buyuk et al., 2022). The EMT progression is regulated by complicated epigenetic mechanisms including EMT-related transcription factors (e.g., snail, slug, and twist), genes (e.g., FOXC2, DYRK2, and PGRMC1), and signaling pathways (e.g., PI3K/Akt, Wnt/*β*-catenin, and ERK1/2) (Lin et al., 2021; Verdura et al., 2021; Debnath et al., 2022). Interestingly, several types of research revealed that EMT progression could be activated by chemotherapeutic agents in some cancer cells. For example, ovarian carcinoma cells exhibited EMT phenotype and stem cell properties along with the increased migration and invasion after the transient cisplatin treatment (Sulaiman et al., 2016). Similarly, low-dose doxorubicin was reported to induce the EMT process and result in a malignant enhancement in gastric cancer cells (Han et al., 2013; Han et al., 2014).

Contactin-1 (*CNTN*-1), a neuronal cell adhesion molecular, was not only involved in the nervous system development including axon guidance, synapse formation, and nerve impulse conduction, but also played a vital role in lymphangiogenesis, lymphatic metastasis, and proliferation of many epithelial malignancies including prostate cancer, hepatocellular cancer, breast cancer, and lung cancer (Bamodu et al., 2020; Cao et al., 2021; Chen et al., 2021; Kandasamy et al., 2022). Additionally, in our prior studies, *CNTN*-1 upregulation was revealed to increase metastasis, invasion, and chemoresistance in lung adenocarcinoma cells with cisplatin resistance (Zhang et al., 2015). Moreover, silencing of *CNTN*-1 expression was recently reported to decrease malignancy and improve the prognosis in patients with gastric cancer by inhibiting EMT progression (Umeda et al., 2022).

Chemotherapeutic agents at low concentrations could facilitate the malignancy of carcinoma cells other than their therapeutic effects (Mottaghi and Abbaszadeh, 2021). However, whether and how low-dose cisplatin promotes the malignant progression of lung adenocarcinoma cells is still unclear. Here, we demonstrated that low-dose cisplatin promoted the malignant progression of lung adenocarcinoma cells through CTN-1 up-regulation-induced activation of the EMT process.

## Materials and Methods

### Cell Culture

Cell line A549 of human lung adenocarcinoma was purchased from the Institute of Biochemistry and Cell Biology, Chinese Academy of Science (Shanghai, China) and cultured in RPMI 1640 medium (Hyclone, United States) supplemented with 10% fetal bovine serum (FBS, Gibco, United States) and 1% penicillin-streptomycin (Hyclone, United States) under standard conditions (37°C, 20% O_2_ and 5% CO_2_). The culture medium was changed every 3 days. When reaching 90% confluence, the cells were passaged using 0.25% trypsin with 0.1% EDTA (Gibco, United States).

### Cell Transfection

The *CNTN*-1 overexpressing lentivirus LV5-*CNTN*-1 was purchased from Genepharma (Shanghai, China) and the full sequence of the recombinant lentiviral vector LV5-*CNTN*-1 could be seen in Supplementary Material S1. Briefly, after being seeded in a 24-well plate for 48 h, A549 cells were transfected with LV5-*CNTN*-1 lentiviral vector in triplicate according to the manufacturer’s protocol, and then fluorescence intensity was observed under the microscope. The successfully transfected cells (A549-*CNTN*-1) were selected *via* 1 g/ml puromycin and cultured for future use.

### Cell Proliferation Assay

Cell proliferation was analyzed using the cell counting kit-8 (CCK-8, Beyotime, China). Briefly, A549 cells were plated in 96-well plates (2000 cells/well) and cultured in the humidified incubator overnight. Then, A549 cells were treated with different concentrations (5, 2, 1, 0.5, and 0 μg/ml) of cisplatin for 24, 48, and 72 h, respectively. At each time point, A549 cells were incubated with 100 μL fresh culture medium and 10 μL CCK-8 solution for 1 h. Cell proliferation potency was reflected by the absorbance value at 450 nm wavelength which was measured using an automatic microplate reader (Bio-rad).

### Cell Apoptosis Assay

A549 cell apoptosis was analyzed by flow cytometry. Briefly, After A549 cells were seeded in Petri dishes (10-cm diameter, 3×10^4^ cells per dish) and grown to 60–70% confluence, A549 cells were incubated with different concentrations (5, 2, 1, 0.5, and 0 μg/ml) of cisplatin for 48 h. After A549 cells were stained with annexin V-FITC/PI stain solution according to the manufacturer’s instructions (Beyotime, China), a flow cytometry assay was performed to detect apoptotic cells which were defined as annexin V-FITC-positive but PI-negative cells, or double positive-stained cells.

### Cell Migration Measurement

Wound-healing assay was used to detect cell migration. Briefly, after A549 cells were seeded in 24-well plates (5×10^5^ cells per well) and cultured overnight, A549 cells were washed with sterile phosphate buffer solution (PBS) and then cultured in the serum-free medium with and without 0.5 μg/ml cisplatin for 24 h. The wound was scratched by sterile pipette tips. Representative images were taken at 0 h and 24 h using a light microscope (Nikon, Japan). Finally, cell migration ability was analyzed using the Image-Pro Plus software (Version 5.1, Media Cybernetics, Inc.).

### Invasion Assay

Cell invasion assay was performed using a transwell chamber with 8 μm pore size polycarbonate membrane in a 24-well plate. Briefly, after the Matrigel (BD, Biosciences) coated on the upper chambers was rehydrated for 1 h at 37°C, 3×10^4^ cells suspended in 200 μL RPMI-1640 medium with different concentrations (0 and 0.5 μg/ml) of cisplatin were added into the upper chambers whereas 500 μL RPMI-1640 medium containing 10% FBS was added into the lower chambers. After incubation for 24 h, cells that passed through the membranes were sequentially washed with PBS, fixed in 4% paraformaldehyde, and then stained with crystal violet dye. Finally, the invasive A549 cells were observed under a light microscope (Nikon, Japan). Cell invasion ability was analyzed using the Image-Pro Plus software (Version 5.1, Media Cybernetics, Inc.).

### *In vitro* Drug Sensitivity Assay

Briefly, cells were seeded in 96-well plates (3×10^3^ cells per well) and incubated with different concentrations of cisplatin (10, 5, 2.5, 1.25, 0.625, and 0 g/ml) for 48 h. Then, cells were incubated with 100 μL fresh culture medium and 10 μL cell counting kit-8 (CCK-8) solution (Beyotime, China) for 1 h. And thereafter, the absorbance value at the wavelength of 450 nm was measured using an automatic microplate reader (Bio-rad). Finally, the 50% inhibitory concentration (IC_50_) was calculated using GraphPad Prism 5.0 software.

### RNA Isolation and Quantitative Real-Time PCR

Briefly, the total RNA was isolated using the Trizol reagent (Invitrogen, United States) according to the manufacturer’s instructions and then 1 μg RNA was reverse transcribed into cDNA using a reverse transcription kit (Roche) according to the manufacturer’s instructions. Primer pairs used for real-time PCR were shown in Table 1. Real-time PCR (qPCR) experiment was performed using a reaction system containing SYBR Green Mix (Agilent Technologies, Santa Clara, United States), cDNA, and primers. The annealing temperature was 62°C and the cycle of gene magnification was 39. Relative expression of target genes was calculated using the 2^−ΔΔCt^ method and normalized to GAPDH.

**TABLE 1:** Primers of target genes.

Gene	Accession number	Forward (5′-3′)	Reverse (5′-3′)
GAPDH	NM_001256799.2	CAG​CCT​CAA​GAT​CAT​CAG​CA	TGT​GGT​CAT​GAG​TCC​TTC​CA
CNTN-1	NM_001256063.1	GCC​CAT​GAC​AAA​GAA​GAA​GC	CGA​CAT​GAT​CCC​AGG​TGA​TT
E-cadherin	NM_001317184.1	GAA​GTG​TCC​GAG​GAC​TTT​GG	CAG​TGT​CTC​TCC​AAA​TCC​TAG​A
N-cadherin	NM_001308176.1	GAG​TCC​ACT​GAG​TAC​CGG​AGA​C	TGT​AGG​TGG​CAA​TCT​CAA​TGT​C
Vimentin	NM_003380.3	AAA​GTG​TGG​CTG​CCA​AGA​AC	AGC​CTC​AGA​GAG​GTC​AGC​AA

### Protein Extraction and Western Blotting

Briefly, after cells were prepared and then washed with PBS, the cells were lysed using 200μl RIPA buffer containing protease inhibitor (Sigma, United States) on the ice for 30 min. After protein concentration was measured using the BCA Protein Assay Kit (Beyotime, Nanjing, China), proteins in each group were separated by SDS-PAGE and then transferred to the PVDF membranes (Millipore, Germany). After that, the membranes were blocked with 5% bovine serum albumin for 1 h at room temperature and incubated with primary antibodies (*CNTN*-1: Proteintech, diluted 1:1000; E-cadherin: Cell Signaling Technology, diluted 1:1000; N-cadherin: Abcam, diluted 1:1000) overnight at 4°C, followed by incubation with corresponding HRP-conjugated secondary antibodies (HRP AffiniPure Goat Anti-Rabbit IgG: EARTHOX, diluted 1:10,000) for 1 h at room temperature. Protein bands on the membrane were detected by SuperSignal West Pico Trial Kit (Thermo, United States) and analyzed using ImageJ software (National Institutes of Health, United States).

### Xenograft Tumorigenicity Experiment

To verify the effects of low-dose cisplatin on A549 cells mediated by *CNTN*-1 in lung adenocarcinoma cells *in vivo*, a xenotransplantation experiment was performed on healthy nude mice (4–5-week old females) purchased from the Animal Laboratory of Xinqiao Hospital affiliated with the Third Military Medical University and housed in a climate-control SPF facility. Briefly, A549 cells treated with or without low-dose (0.5 μg/ml) cisplatin was injected into the right flank of nude mice (100 μL, 1 × 10^7^ cells per animal). During the next 30 days, tumor volume was measured every 3 days. At the end of the experiment, all animals were sacrificed and xenograft tumors were excised to detect *CNTN*-1 and EMT biomarkers *via* immunohistochemistry or Western blot. All animal experiments were approved by Zhonghong Boyuan Biotechnology Co., Ltd. [SYXK (GAN) 2020-0001].

### Statistical Analysis

All numerical data are expressed as means ± SD and each experiment was performed at least in triplicate in this study. After the homogeneity test for variance was finished, comparisons between two groups were performed by Independent-Samples t test, whereas comparisons between multiple groups were performed by one-way analysis of variance (ANOVA) using SPSS 13.0 software. A significant difference was indicated when the *p*-value < 0.05.

## Results

### Effects of Low-Dose Cisplatin on the Viability of A549 Cells

**FIGURE 1:**
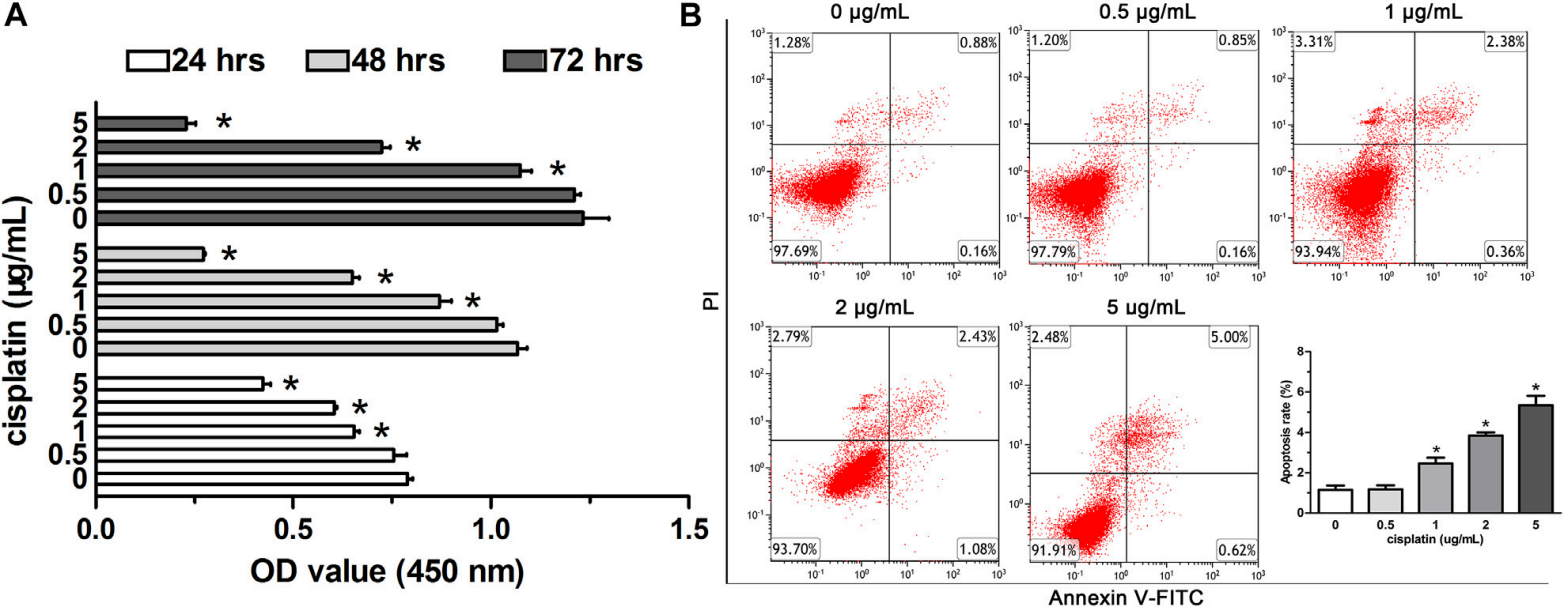
Effects of low-dose cisplatin treatment on the viability of A549 cells. **(A)** Cell proliferation of A549 cells was detected by the CCK-8 method after incubation with different concentrations of cisplatin (5, 2, 1, 0.5, 0 g/mL) for 24, 48, and 72 h, respectively. As shown in **(A)**, the proliferation of A549 cells incubated with low-dose (0.5 μg/ml) cisplatin was similar to that of A549 cells incubated without cisplatin during the whole experiment duration (*p > 0.05). **(B)** Apoptosis of A549 cells was assessed with Flow cytometry analysis after being treated with different concentrations of cisplatin (5, 2, 1, 0.5, 0 g/mL). PI staining was indicated as red fluorescence and Annexin V as green fluorescence. As shown in **(B)**, apoptosis of A549 cells was not affected by the low-dose cisplatin (0.5 μg/ml) compared with that of controls (A549 cells treated without cisplatin, *p > 0.05). Each experiment was conducted in triplicate and the data were expressed as mean ± SD.

To study the viability of A549 cells affected by low-dose cisplatin, the cells were treated with the gradient cisplatin concentrations (5, 2, 1, 0.5, and 0 μg/ml) and the results showed that the proliferation of A549 cells was attenuated with elevating cisplatin concentration (from 1 to 5 μg/ml) and with extending experiment duration (from 24 to 72 h). However, the proliferation of A549 cells incubated with low-dose (0.5 μg/ml) cisplatin was similar to that of A549 cells incubated without cisplatin during the whole experiment duration (Figure 1A). Additionally, compared with that of controls (A549 cells incubated with cisplatin-free medium), apoptosis of A549 cells treated with a higher dose of cisplatin (from 1 to 5 μg/ml) was enhanced except for low-dose cisplatin (0.5 μg/ml) which did not have obvious effects on apoptosis of A549 cells (Figure 1B). Because there was no significant difference in proliferation and apoptosis between A549 cells treated with 0.5 μg/ml cisplatin and controls, 0.5 μg/ml cisplatin was used as the low dose in the following experiment.

### Low-Dose Cisplatin Transient Treatment Induced EMT Phenotype in A549 Cells

**FIGURE 2:**
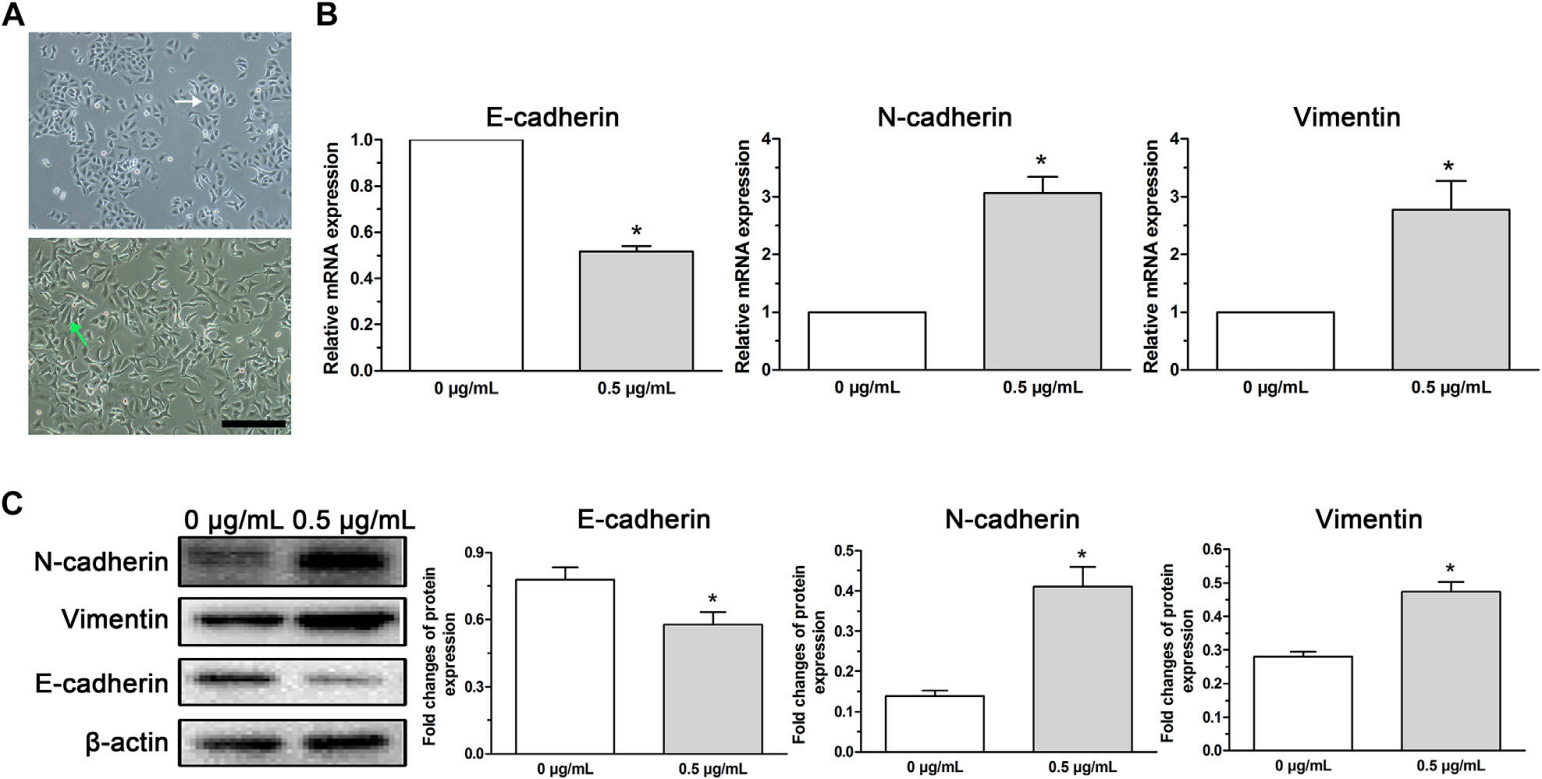
EMT phenotype cells induced in A549 cells by low-dose cisplatin transient treatment. **(A)** Morphological observation of A549 cells under a light microscope. A549 cells incubated with low-dose cisplatin (0.5 μg/ml) presented a spindle-like shape and irregular cell arrangement (magnification: ×100, scale bar = 100 um). **(B,C)** EMT-related markers (E-cadherin, N-cadherin, and vimentin) were evaluated by RT-PCR and Western blot, respectively. As shown in **(B,C)**, mesenchymal markers (N-cadherin and vimentin) were upregulated whereas epithelial marker (E-cadherin) was downregulated in A549 cells treated with low-dose (0.5 μg/ml) cisplatin compared with that of controls both at the mRNA and protein levels (*p < 0.01). Each experiment was conducted in triplicate and the data was expressed as mean ± SD.

After low-dose (0.5 μg/ml) cisplatin transient treatment for 48 h and the following culture for 24 h in cisplatin-free medium, cell death was detected but the survived cells displayed morphological changes characterized by a spindle-like shape and irregular cell arrangement (Figure 2A), which were similar to EMT phenotype. The cells with these morphological changes were used to perform cell function experiments and molecular experiments.

To analyze whether the morphological changes of A549 cells induced by low-dose (0.5 μg/ml) cisplatin was associated with EMT phenotype, EMT-related markers (E-cadherin, N-cadherin, and vimentin) were detected. Results showed that mesenchymal markers (N-cadherin and vimentin) were upregulated whereas epithelial marker (E-cadherin) was downregulated in A549 cells treated with low-dose (0.5 μg/ml) cisplatin compared with that of controls both at the mRNA and protein levels (Figures 2B, C), suggesting that low-dose cisplatin transient treatment activated EMT process in A549 cells.

### Low-Dose Cisplatin Treatment Promoted EMT Progression of A549 Cells

**FIGURE 3:**
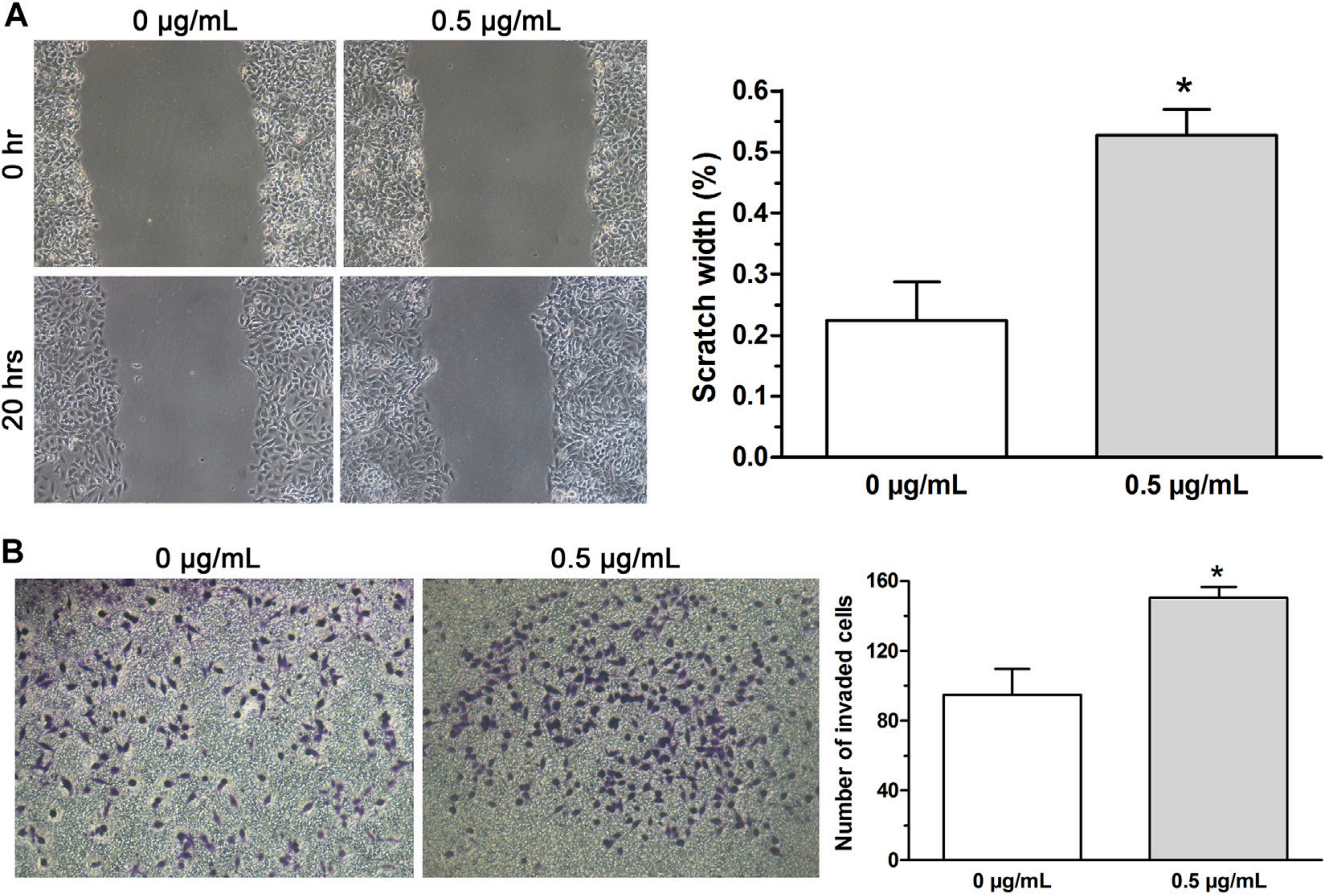
EMT progression of A549 cells promoted by low-dose cisplatin treatment. **(A,B)** Migration and invasion of A549 cells were measured by wound healing assay and transwell invasion assay, respectively. The migration and invasive ability of A549 cells treated with low-dose (0.5 μg/ml) cisplatin was increased compared with that of controls (*p < 0.01). All pictures were photographed at a magnification of ×100, scale bar = 100 um. Each experiment was conducted in triplicate and the data were expressed as mean ± SD.

Previous reports revealed that the activation of EMT process renders cytoskeleton remolding of cancer cells followed by the acquirement of new characteristics including the enhanced metastasis and invasion (de Araújo et al., 2022; Meng et al., 2022). Therefore, in this study, wound-healing assay and invasion assay were conducted to examine cell migration and invasion and the data displayed that both migration (Figure 3A) and invasive ability (Figure 3B) of A549 cells treated with low-dose (0.5 μg/ml) cisplatin were increased compared with that of controls. Together, the findings indicated that low-dose cisplatin treatment could promote EMT progression of A549 cells.

### EMT Progression in A549 Cells Induced by Low-Dose Cisplatin Related With Upregulated *CNTN*-1

**FIGURE 4:**
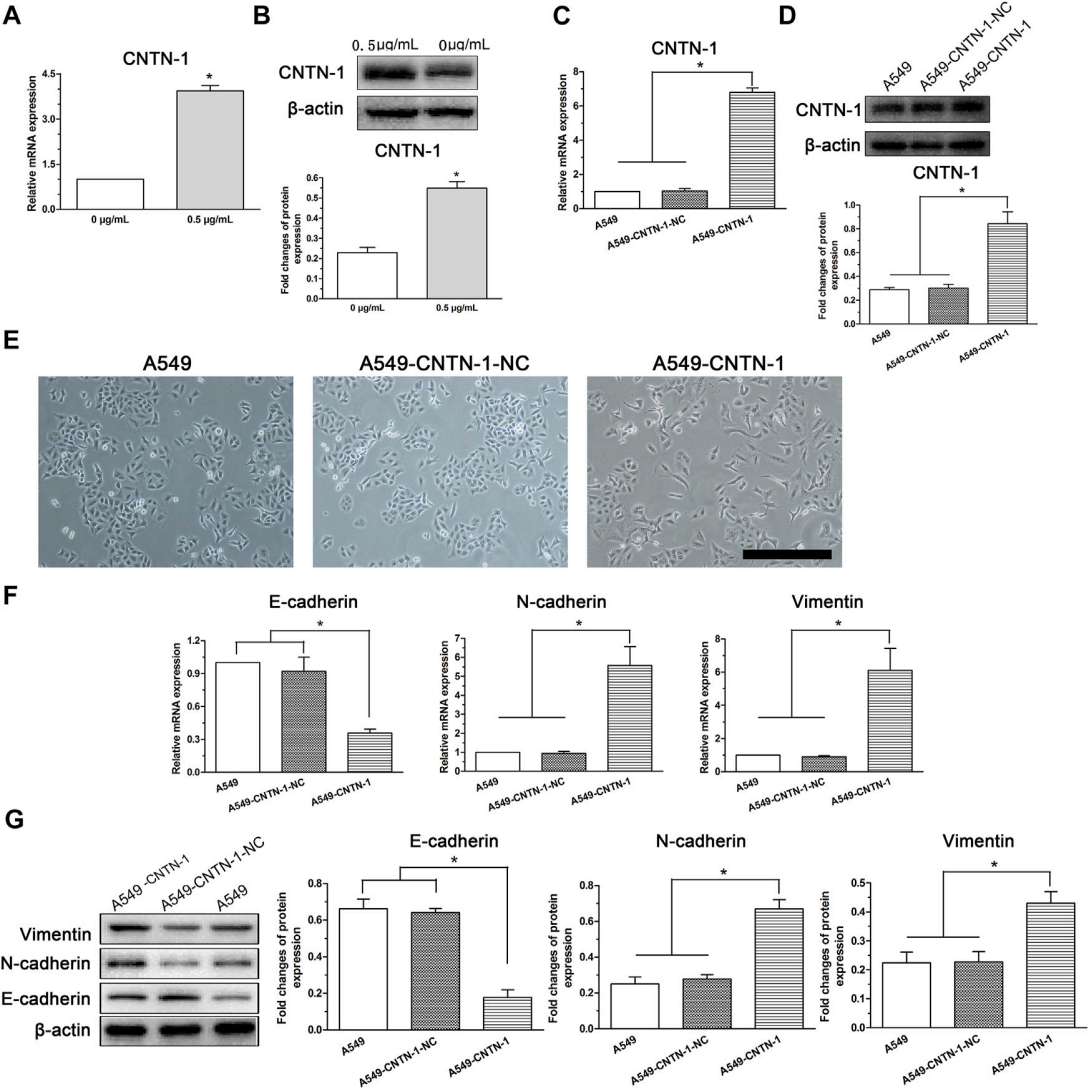
EMT progression of A549 cells induced by low-dose cisplatin treatment related to *CNTN*-1 expression. **(A,B)** *CNTN*-1 was evaluated by RT-PCR and Western blot, respectively. *CNTN*-1 was upregulated in A549 cells treated with low-dose cisplatin (0.5 μg/ml) compared with that of controls (*p < 0.05). **(C,D)** *CNTN*-1 overexpression in A549-*CNTN*-1 cells was verified by RT-PCR and Western blot, respectively. *CNTN*-1 was successfully overexpressed in A549-*CNTN*-1 cells both at gene and protein levels (*p < 0.01). **(E)** EMT phenotype observed in A549-*CNTN*-1 cells (magnification: ×100, scale bar = 100 um). **(F,G)** EMT-related markers were detected in A549-*CNTN*-1 cells. Both N-cadherin and vimentin were increased whereas E-cadherin was decreased both at gene and protein levels in A549-*CNTN*-1 cells (*p < 0.01). Each experiment was conducted in triplicate and the data were expressed as mean ± SD.

Our previous study revealed that *CNTN*-1 upregulation in lung adenocarcinoma patients was correlated with lymphatic invasion during platinum-based chemotherapy (Zhang et al., 2015). Moreover, *CNTN*-1 downregulation was recently reported to inhibit EMT progression in gastric cancer cells (He et al., 2022). Therefore, to investigate whether *CNTN*-1 plays a role in the low-dose cisplatin treatment-induced EMT process and malignant progression in A549 cells, *CNTN*-1 in A549 cells treated with or without low-dose cisplatin was measured. As shown in Figures 4A,B, and *CNTN*-1 in A549 cells incubated with low-dose (0.5 μg/ml) cisplatin medium was higher than that of A549 cells incubated without cisplatin.

To determine the role of *CNTN*-1 in mediating the EMT process in A549 cells, after *CNTN*-1 was overexpressed in A549 cells (A549-*CNTN*-1, Figures 4C,D), the morphological changes were detected and the data revealed that EMT phenotype was observed in A549-*CNTN*-1 cells compared with that of controls (A549-*CNTN*-1-NC, Figure 4E), verified by the increased expression of N-cadherin and vimentin and the decreased expression of E-cadherin both at gene and protein levels (Figures 4F,G), which strongly suggests that *CNTN*-1 upregulation induced by low-dose cisplatin treatment promoted EMT phenotype of A549 cells.

**FIGURE 5:**
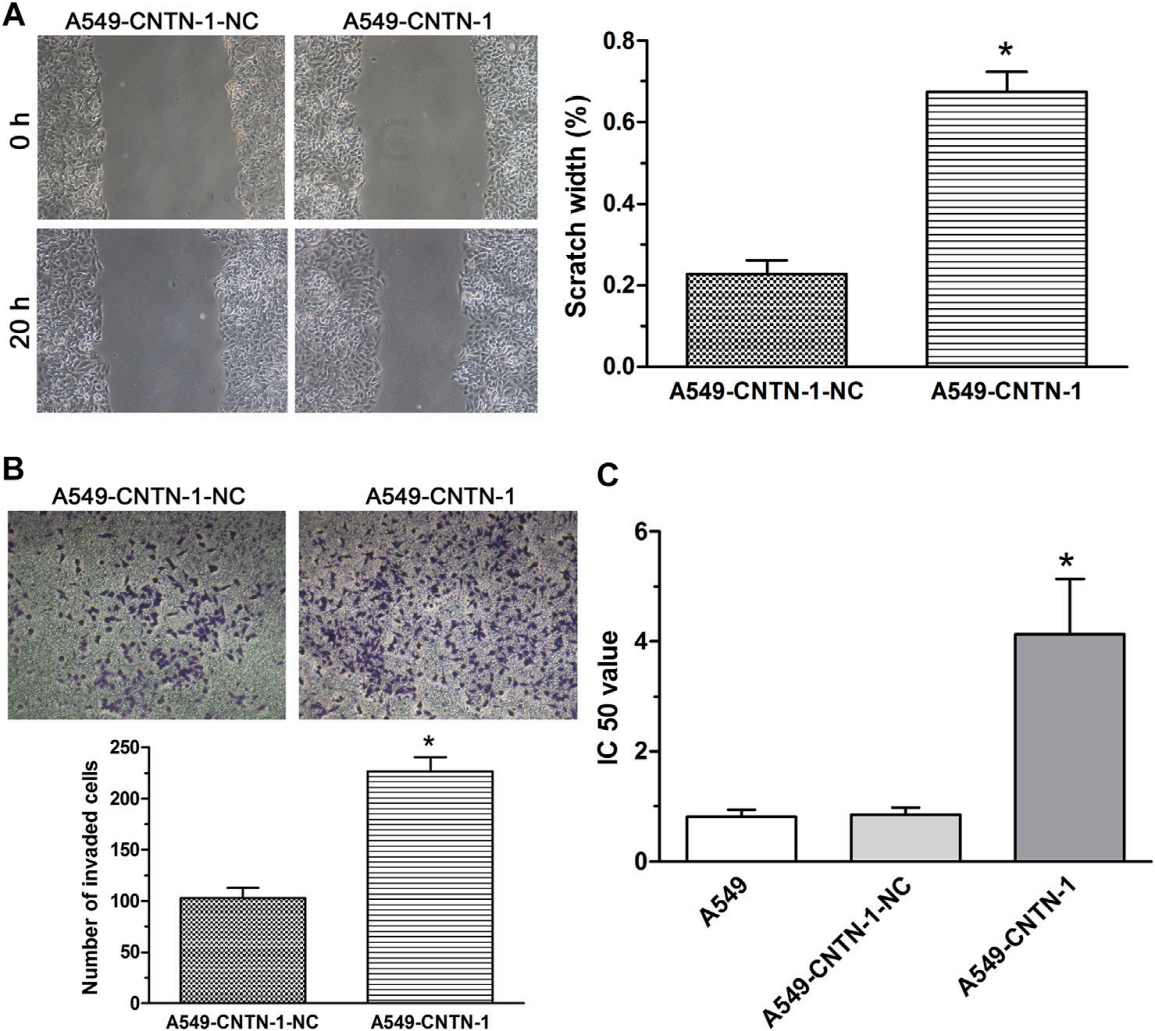
EMT progression promoted by *CNTN*-1 overexpression. **(A,B)** Migration and invasion of A549-*CNTN*-1 cells were measured by wound healing assay and transwell invasion assay, respectively. Both migration and invasion of A549-*CNTN*-1 cells were dramatically enhanced compared with that of controls (*p < 0.01). **(C)** Drug sensitivity of A549-*CNTN*-1 cells evaluated by CCK-8 method. A549-*CNTN*-1 cells were more tolerant to cisplatin than A549 cells (*p < 0.01). All pictures were photographed at a magnification of ×100, scale bar = 100 um. Each experiment was conducted in triplicate and the data were expressed as mean ± SD.

Because the aberrant activation of the EMT process enhanced malignant progression (including metastasis, invasion, and chemoresistance) of various kinds of cancer cells (Girisa et al., 2021; Cook and Wrana, 2022), to evaluate the association of *CNTN*-1 with malignant progression *via* EMT, the wound-healing assay, invasion assay, and drug sensitivity assay were carried out and the data showed that *CNTN*-1 overexpression dramatically enhanced the migration (Figure 5A), invasion (Figure 5B), and tolerance to cisplatin (A549-*CNTN*-1 vs. A549-*CNTN*-1-NC, Figure 5C). These findings indicated that *CNTN*-1 upregulation promoted EMT phenotype, which enhanced the malignant progression of A549 cells.

### *CNTN*-1 Upregulation Induced by Low-Dose Cisplatin Activated EMT Process *in vivo*

**FIGURE 6:**
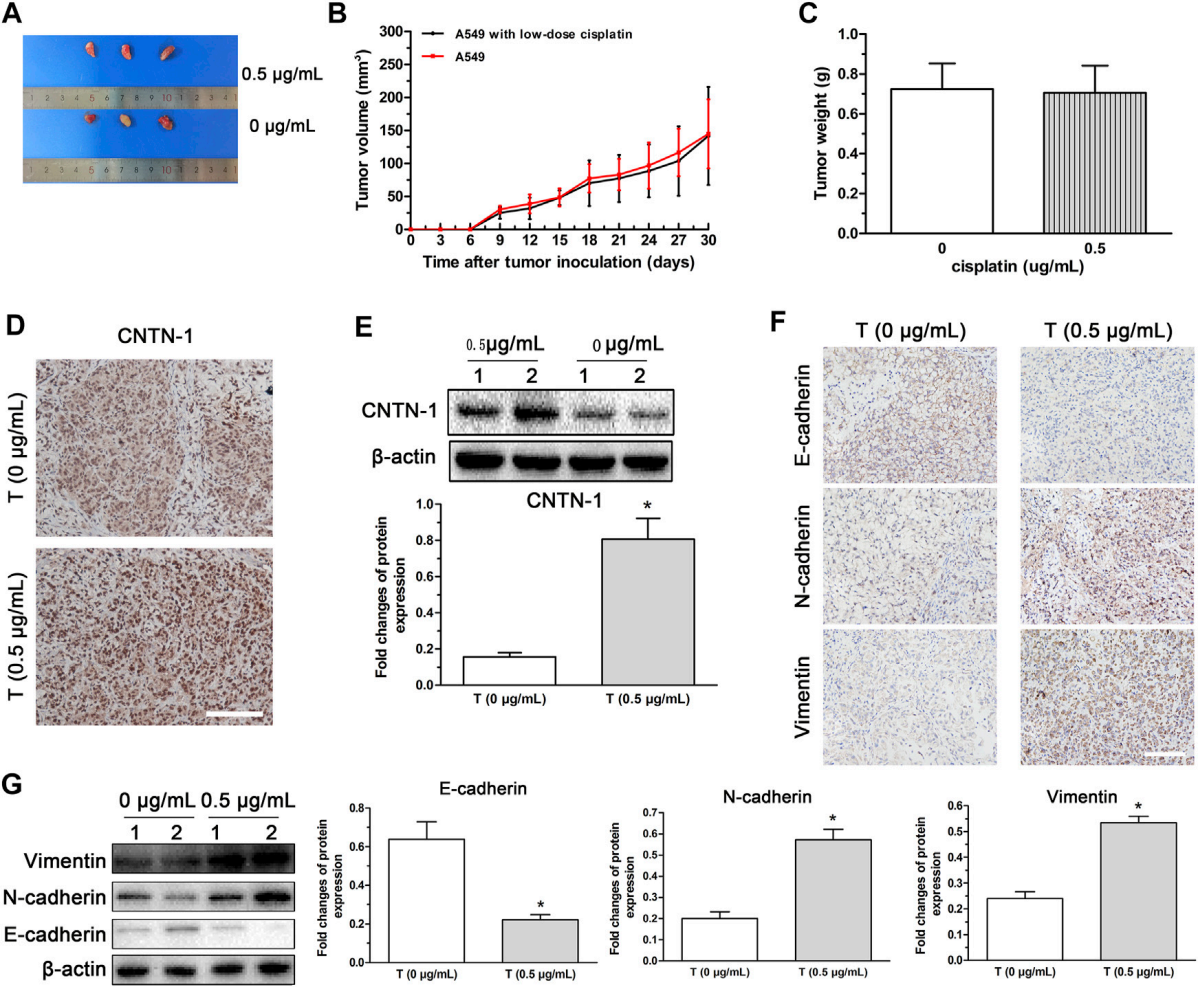
*CNTN*-1 upregulation and EMT process induced in xenograft tumors developed with A549 cells treated with low-dose cisplatin. **(A)** Tumors developed with A549 cells treated with low-dose cisplatin (0.5 g/mL) and without cisplatin (control), respectively. **(B)** Growth curves of tumors. No significant difference in volume was observed between tumors developed with A549 cells treated with low-dose cisplatin (0.5 g/mL) and controls. **(C)** Tumor weight. No significant difference of weight was observed between tumors developed by A549 cells treated with low-dose cisplatin (0.5 g/mL) and controls (*p > 0.05). **(D–G)** *CNTN*-1 and EMT-related markers (E-cadherin, N-cadherin, and vimentin) of tumors measured by Western blot and immunohistochemistry, respectively. Both *CNTN*-1 and mesenchymal markers (N-cadherin and vimentin) were significantly upregulated whereas epithelial marker (E-cadherin) was downregulated in the xenograft tumor developed by A549 cells treated with low-dose cisplatin compared with that of controls (*p < 0.05). All pictures were photographed at a magnification of ×100, scale bar = 100 um. Each experiment was conducted in triplicate and the data were expressed as mean ± SD.

To verify the effects of low-dose cisplatin on *CNTN*-1 expression and activation of EMT progression *in vivo*, a nude mice xenotransplantation model was developed by injection with A549 cells treated with low-dose (0.5 μg/ml) cisplatin or without cisplatin (controls). As shown in Figures 6A–C, no significant differences in the volume and weight were observed between tumors developed with A549 cells treated with low-dose cisplatin and controls. Results of immunohistochemistry and Western blot assay showed that *CNTN*-1 was increased in the xenograft tumor of A549 cells treated with low-dose cisplatin compared with that of controls (Figures 6D,E). Furthermore, both N-cadherin and vimentin were increased whereas E-cadherin was significantly decreased in the xenograft tumor of A549 cells treated with low-dose cisplatin compared with that of controls (Figures 6F,G), which strongly confirmed that EMT was activated by *CNTN*-1 upregulation induced by low-dose cisplatin.

## Discussion

Platinum-based chemotherapy is the major treatment of advanced lung adenocarcinoma with epithelial growth factor receptor (EGFR) wide-type and ineffective immunotherapy in clinical practice (Deng et al., 2022). Despite the effectively initial responses to chemotherapy, a majority of patients ultimately succumbed to malignant progression, leading to a dismal prognosis (Giacomini et al., 2020). In recent years, accumulating evidence has shown that chemotherapeutic agents have the potential to promote the malignant progression of several carcinoma cells but not their efficiency in eliminating cancer cells (Latifi et al., 2011; Han et al., 2013; Wang et al., 2014; Liu et al., 2015; Lohan-Codeço et al., 2022). Improved understanding of the mechanisms of chemotherapeutic drug-induced tumor progression will be important to provide valuable information to clinical tumor chemotherapy.

The aberrant activation of the EMT process accelerates malignancy and drug tolerance of several epithelial carcinomas (Song et al., 2022). EMT progression is regulated by encoding genes, transcription factors, and Circulating exosomes (Marimuthu et al., 2021). Subsequent research showed that some medicines also had a function on EMT progression instead of their therapeutic action. For example, EGFR tyrosine kinase inhibitors (EGFR-TKIs, e.g., gefitinib and erlotinib) could induce the occurrence of the EMT process and in turn influence their malignant behavior even drug resistance *by* regulating related signaling pathways and transcription factors (Huang and Fu, 2015). Similarly, chemotherapeutics also had the function in tumor progression otherwise therapeutic effects *via* modulating EMT activation. For example, the appropriate dose of doxorubicin could activate the EMT progress and in turn, promote the malignancy in gastric cancer cells *via β*-catenin signaling (Wang et al., 2020). In addition, cisplatin treatment could induce the EMT phenotype and facilitate malignant progression even drug resistance in ovarian carcinoma cells and prostate cancer cells by several transcription factors such as Snail, Slug, and metalloproteinase 9 (Latifi et al., 2011; Liu et al., 2015). To evaluate the association of low-dose cisplatin treatment with the EMT process of lung adenocarcinoma, we first examined the morphology of A549 cells after the transient low-dose cisplatin treatment. Results showed that A549 cells treated with transient low-dose cisplatin lost their intrinsic epithelial-like cell polarity whereas exhibited spindle-shaped mesenchymal morphology and that A549 cells treated with transient low-dose cisplatin had upregulated mesenchymal markers (N-cadherin and vimentin) expression and downregulated epithelial marker (E-cadherin) expression, indicating that transient low-dose cisplatin can induce EMT process in A549 cells. Because the aberrant activation of the EMT process was reported to promote tumor progression (Aleksakhina et al., 2019; Gu et al., 2021), to analyze whether the EMT process induced by the low-dose cisplatin can further enhance the malignant process of A549 cells, migration and invasion were compared between A549 cells treated with low-dose cisplatin and without cisplatin. The comparison demonstrated that metastasis and invasion of A549 cells treated with cisplatin were increased significantly compared that of A549 cells treat without cisplatin, indicating that transient low-dose cisplatin treatment promoted the malignant progression of A549 cells.

*CNTN*-1, located on chromosome 12q11-q12 and a neuronal cell adhesion molecules of the immunoglobulin superfamily, participates in nervous system development (Su et al., 2006). Additionally, *CNTN*-1 was also reported to promote the malignant progression of cancer cells as a downstream molecule of the VEGF-C/Flt-4 axis (Zhou et al., 2015; Liang et al., 2020). In our previous studies, *CNTN*-1 expression was found to be positively correlated with lymphatic invasion of lung adenocarcinoma patients who received adjuvant cisplatin- or carboplatin-based treatment after surgery (Chen et al., 2015), suggesting that the *CNTN*-1 upregulation may be induced by platinum-based chemotherapy. To prove the hypothesis, *CNTN*-1 expression was analyzed in A549 cells treated with and without low-dose cisplatin. Importantly, *CNTN*-1 expression was found to be higher in A549 cells treated with cisplatin than in A549 cells treated without cisplatin. Moreover, the upregulated *CNTN*-1 was found to promote EMT progression, verified by changes in cellular morphology, abnormal expression of EMT-related molecules (E-cadherin, N-cadherin, and vimentin), and increased metastasis and invasion of A549-*CNTN*-1 cells. Taken together, these results indicated that low-dose cisplatin-induced EMT and malignant progression of A549 cells were positively regulated by *CNTN*-1 upregulation, which was consistent with the previous report that *CNTN*-1 upregulation enhanced cancer metastasis and invasion *via* EMT alteration in gastric cancer (42).

To prove the effects low-dose of cisplatin on the EMT process *in vivo*, a nude xenotransplantation mice model was established by injection with A549 cells treated with or without low-dose (0.5 μg/ml) cisplatin. Similar to the findings *in vitro*, *CNTN*-1 was increased and the EMT process was activated in xenograft tumor developed with A549 cells treated with low-dose cisplatin compared with that of A549 cells treated without cisplatin, confirming that low-dose cisplatin could upregulate *CNTN*-1 which activated EMT process subsequently. However, because the xenograft mouse model was not suitable to observe tumor metastasis, the *in vivo* tumor metastasis was not investigated in this study.

To our knowledge, this is the first study reporting that *CNTN*-1 upregulation induced by the low-dose cisplatin activated EMT process and thus promoted malignant progression in lung adenocarcinoma cells. The findings provided new evidence that platinum-based chemotherapy could facilitate malignancy of carcinoma cells *via* activation of the EMT process by CNTN-1 overexpression other than their therapeutic effects, indicating that inhibiting the expression of CNTN-1 may reverse the EMT process and in turn, enhance the efficiency of platinum-based chemotherapy.

## Data Availability

The original contributions presented in the study are included in the article/Supplementary Material, further inquiries can be directed to the corresponding author.
