# Supplementary material for: CNTN-1 Upregulation Induced by Low-Dose Cisplatin Promotes Malignant Progression of Lung Adenocarcinoma Cells via Activation of Epithelial-Mesenchymal Transition
Source: Front Genet. 2022 May 27;13:891665. doi: 10.3389/fgene.2022.891665 (PMC9196332; doi:10.3389/fgene.2022.891665)
Supplement: Supplementary file 1 [file DataSheet1.doc]

**Supplementary Material 1:** The full sequence of contactin-1 (CNTN-1).

| Full sequence of CNTN-1 |
| --- |
| atgaaaatgtggttgctggtcagtcatcttgtgataatatctattactacctgtttagcagagtttacatggtatagaagatatggtcatggagtttctgaggaagacaaaggatttggaccaatttttgaagagcagccaatcaataccatttatccagaggaatcactggaaggaaaagtctcactcaactgtagggcacgagccagccctttcccggtttacaaatggagaatgaataatggggacgttgatctcacaagtgatcgatacagtatggtaggaggaaaccttgttatcaacaaccctgacaaacagaaagatgctggaatatactactgtttagcatctaataactacgggatggtcagaagcactgaagcaaccctgagctttggatatcttgatcctttcccacctgaggaacgtcctgaggtcagagtaaaagaagggaaaggaatggtgcttctctgtgaccccccataccattttccagatgatcttagctatcgctggcttctaaatgaatttcctgtatttatcacaatggataaacggcgatttgtgtctcagacaaatggcaatctctacattgcaaatgttgaggcttccgacaaaggcaattattcctgctttgtttccagtccttctattacaaagagcgtgttcagcaaattcatcccactcattccaatacctgaacgaacaacaaaaccatatcctgctgatattgtagttcagttcaaggatgtatatgcattgatgggccaaaatgtgaccttagaatgttttgcacttggaaatcctgttccggatatccgatggcggaaggttctagaaccaatgccaagcactgctgagattagcacctctggggctgttcttaagatcttcaatattcagctagaagatgaaggcatctatgaatgtgaggctgagaacattagaggaaaggataaacatcaagcaagaatttatgttcaagcattccctgagtgggtagaacacatcaatgacacagaggtggacataggcagtgatctctactggccttgtgtggccacaggaaagcccatccctacaatccgatggttgaaaaatggatatgcgtatcataaaggggaattaagactgtatgatgtgacttttgaaaatgccggaatgtatcagtgcatagctgaaaacacatatggagccatttatgcaaatgctgagttgaagatcttggcgttggctccaacttttgaaatgaatcctatgaagaaaaagatcctggctgctaaaggtggaagggtgataattgaatgcaaacctaaagctgcaccgaaaccaaagttttcatggagtaaagggacagagtggcttgtcaatagcagcagaatactcatttgggaagatggtagcttggaaatcaacaacattacaaggaatgatggaggtatctatacatgctttgcagaaaataacagagggaaagctaatagcactggaacccttgttatcacagatcctacgcgaattatattggccccaattaatgccgatatcacagttggagaaaacgccaccatgcagtgtgctgcgtcctttgatcctgccttggatctcacatttgtttggtccttcaatggctatgtgatcgattttaacaaagagaatattcactaccagaggaattttatgctggattccaatggggaattactaatccgaaatgcgcagctgaaacatgctggaagatacacatgcactgcccagacaattgtggacaattcttcagcttcagctgaccttgtagtgagaggccctccaggccctccaggtggtctgagaatagaagacattagagccacttctgtggcacttacttggagccgtggttcagacaatcatagtcctatttctaaatacactatccagaccaagactattctttcagatgactggaaagatgcaaagacagatcccccaattattgaaggaaatatggaggcagcaagagcagtggacttaatcccatggatggagtatgaattccgcgtggtagcaaccaatacactgggtagaggagagcccagtataccatctaacagaattaaaacagacggtgctgcaccaaatgtggctccttcagatgtaggaggtggaggtggaagaaacagagagctgaccataacatgggcgcctttgtcaagagaataccactatggcaacaattttggttacatagtggcatttaagccatttgatggagaagaatggaaaaaagtcacagttactaatcctgatactggccgatatgtccataaagatgaaaccatgagcccttccactgcatttcaagttaaagtcaaggccttcaacaacaaaggagatggaccttacagcctagtagcagtcattaattcagcacaagacgctcccagtgaagccccaacagaagtaggtgtaaaagtcttatcatcttctgagatatctgttcattgggaacatgttttagaaaaaatagtggaaagctatcagattcggtattgggctgcccatgacaaagaagaagctgcaaacagagttcaagtcaccagccaagagtactcggccaggctcgagaaccttctgccagacacccagtattttatagaagtcggggcctgcaatagtgcagggtgtggacctccaagtgacatgattgaggctttcaccaagaaagcacctcctagccagcctccaaggatcatcagttcagtaaggtctggttcacgctatataatcacctgggatcatgtcgttgcactatcaaatgaatctacagtgacgggatataaggtactctacagacctgatggccagcatgatggcaagctgtattcaactcacaaacactccatagaagtcccaatccccagagatggagaatacgttgtggaggttcgcgcgcacagtgatggaggagatggagtggtgtctcaagtcaaaatttcaggtgcacccaccctatccccaagtcttctcggcttactgctgcctgcctttggcatccttgtctacttggaattctga |
